# Immune Cell Proinflammatory Microenvironment and Androgen-Related Metabolic Regulation During Benign Prostatic Hyperplasia in Aging

**DOI:** 10.3389/fimmu.2022.842008

**Published:** 2022-03-21

**Authors:** Dehong Cao, Ruonan Sun, Lei Peng, Jinze Li, Yin Huang, Zeyu Chen, Bo Chen, Jin Li, Jianzhong Ai, Lu Yang, Liangren Liu, Qiang Wei

**Affiliations:** ^1^ Department of Urology, West China Hospital, Sichuan University, Chengdu, China; ^2^ Institute of Urology, West China Hospital, Sichuan University, Chengdu, China; ^3^ West China School of Medicine, Sichuan University, Chengdu, China; ^4^ Department of Urology, Nanchong Central Hospital, The Second Clinical College, North Sichuan Medical College (University), Nanchong, China

**Keywords:** benign prostatic hyperplasia, pathogenesis, disease progression, aging, inflammation

## Abstract

To review the role of inflammation in the occurrence and development of benign prostatic hyperplasia (BPH), we searched PubMed for the latest published articles up to February 2021 using the following key words: “benign prostatic hyperplasia”, “inflammation”, “pathogenesis” and “disease development”. Articles were obtained and reviewed to provide a systematic review of the current progress of the role of inflammation in the pathogenesis and progression of BPH. Inflammation contributes to the initiation and maintenance of unregulated cell proliferation and is closely related to the occurrence and development of BPH. Its action pathways include tissue damage and subsequent chronic healing, autoimmunity, and coaction with androgens. During the progression of inflammation, macrophages, interleukin-8 (IL-8), interleukin-1 (IL-1) and other inflammatory-related substances aggregate locally and cause BPH through various biochemical pathways. At the same time, BPH can also counteract inflammation to expand its scope and aggravate the situation. Inflammation can independently affect the development of BPH in a variety of ways, and it can also interact with androgens. In the course of treatment, early intervention in the occurrence and development of inflammation in prostate tissue can slow down the progression of BPH. The combination of standard therapies and anti-inflammatory measures may provide valuable new ideas for the treatment of BPH.

## Introduction

Benign prostatic hyperplasia (BPH) is a common disease in middle-aged and elderly men. The clinical manifestation is the formation of prostatic transition zone hyperplasia and nodules, which is the most common cause of lower urinary tract symptoms. The disease progresses slowly, and it takes a long time to evolve from the initial tissue lesions to the appearance of clinical symptoms. The cellular and molecular mechanisms of its pathogenesis have not yet been fully elucidated. In traditional research (1), changes in androgen levels and tissue remodeling caused by aging are generally considered to be the main determinants of prostate hyperplasia. In prostate epithelial and stromal cells, testosterone produced by the testis diffuses into the prostate epithelium and stromal cells. In stromal cells, most testosterone is converted to dihydrotestosterone, which can act in an autocrine manner in stromal cells or diffuse into nearby epithelial cells, acting in a paracrine manner (2). It has a high affinity for androgen receptor (AR) (2–4), which can be adjusted by the activity of several growth factors or their receptors. The expression of AR thus has an effect on cell proliferation in epithelial and stromal cells (5). However, in recent studies, inflammation has shown a potential role in the pathogenesis and progression of BPH (6). A recent histological and biochemical study showed (7) that inflammation is more frequently found in prostate patients, and patients with prostatitis have a higher International Prostate Symptom Score (I-PSS) and larger prostate volume. Examination of 167 prostates showed that inflammation was more common in glands with BPH than in glands without BPH (75% *vs* 50%, p<0.01). Another study conducted a 4-year longitudinal evaluation of 4109 men and confirmed that chronic inflammation is associated with the occurrence and development of BPH (8). Tissue damage related to inflammation and subsequent chronic tissue healing may be an important reason for the inflammatory proliferation of BPH prostate tissue cells. With the progression of inflammation, inflammation-related substances in the prostate accumulate locally, which can increase the growth rate of cells. The onset of BPH can even aggravate the inflammatory response. The two are in a mutually promoting pathological state. Based on the evidence described above, this article reviews the role of inflammation in the pathogenesis and progression of BPH.

## Occurrence of BPH

BPH can be defined as the excessive proliferation of prostate stromal cells and epithelial cells caused by complex cellular changes. In molecular cytology, the pathogenesis of BPH is not yet fully understood. Stromal cells, immune cells and epithelial cells are important components of proliferative prostate tissue. The occurrence of BPH is the combined effect of different factors on the three components (9). Histologically, BPH can be defined as an increase in the volume of the prostate transition zone associated with tissue remodeling, which involves epithelial tissue and fibromuscular matrix, showing a certain androgen dependence (10, 11). Compared with normal prostate tissue, the balance between growth and apoptosis of stromal cells in hyperplastic nodules is broken, eventually leading to an increase in stromal volume. The cellular and molecular mechanisms of the abnormal proliferation of the abovementioned cells have not been fully elucidated. Traditionally, the excessive proliferation of cells in the prostate is related to the expression and activity of androgens and their receptors. Recent clinical evidence (3, 12, 13) shows that, for uncontrolled cell proliferation, the presence of inflammation contributes to the initiation and maintenance of proliferation, which is closely related to the occurrence and development of BPH. Its action pathways include tissue damage and subsequent chronic healing, autoimmunity, and synergy with the androgens.

## The Role of Inflammation in BPH

It has been confirmed that prostate tissue can be directly affected by androgens, which can also work in the progression of BPH. There has been clinical evidence that taking 5α-reductase inhibitors (5AR-I) can reduce the dihydrotestosterone (DHT) concentration in prostate tissue, thereby preventing further development of BPH. Compared with dihydrotestosterone, testosterone is less able to bind to AR and stimulate BPH progression. However, up to 10% of patients still show clinical progression (14). In addition, the risk of developing BPH increases with age. However, testosterone levels gradually decrease with age (15), which is likely to mean that hormonal factors alone may not fully explain the pathogenesis of BPH.

A review of resected tissue from prostates that had symptoms deemed severe enough for surgery revealed the universal presence of varying histologic inflammation in a population with a high prevalence of preoperative catheterization (16). The REDUCE trial reported the results of prostate biopsy on 8824 patients and found that 77.6% of the samples had chronic inflammation (17). Another group of researchers conducted multivariate statistical analysis and found that BPH patients with prostatitis were 7 times more likely to have lower urinary tract symptoms than those without inflammation (HR: 6.84; 95% CI: 4.5-11.78; P <0.0001) (17, 18). Rober and his team studied 282 specimens from patients undergoing BPH surgery (19). They found that the average prostate volume of BPH patients in the severe inflammation group was 15 ml higher than that in the mild inflammation group (P=0.002) (19). In addition, the average I-PSSs of the low inflammation group and the high inflammation group were 12 and 21, respectively (P=0.02) (19). Therefore, inflammation will lead to the occurrence and development of BPH. To some extent, the degree of inflammation is positively correlated with prostate volume.

In addition to the severity of immune inflammation, the distribution of inflammation in prostate epithelial and stromal tissue is also an important factor affecting the clinical symptoms of BPH patients. Some scholars divided the prostate tissue specimens of 179 surgical patients into a stromal group and a nonstromal group according to the different parts of prostate inflammation and analyzed the influence of different parts of inflammation on the clinical symptoms of BPH patients. The results suggest that BPH patients in the stromal group have a larger prostate volume, a higher incidence of acute urinary retention, and a smaller maximum urine flow rate than those in the nonstromal group. The lower urinary tract symptoms of patients with benign prostatic hyperplasia will continue to worsen as the degree of inflammation in the stroma increases (20). Distinguishing whether inflammation occurs in stroma or epithelial tissue is helpful for understanding the progression of BPH with immunological inflammation and lower urinary tract symptoms. It also played a guiding role in clinical treatment. Timely and effective surgical treatment may be an early treatment for BPH patients with stromal inflammation (21).

In the past few years, the role of inflammation in the pathogenesis of BPH was once thought to support the process of fiber muscle growth. Some researchers believe that tissue damage related to inflammation and subsequent chronic tissue healing may be an important cause of the inflammatory proliferation of BPH prostate tissue cells (12). Inflammation causes cell and DNA damage, promotes cell replacement and creates a tissue microenvironment rich in cytokines and growth factors, thereby promoting cell proliferation and causing hyperplasia during tissue repair (13).

**Figure 1:**
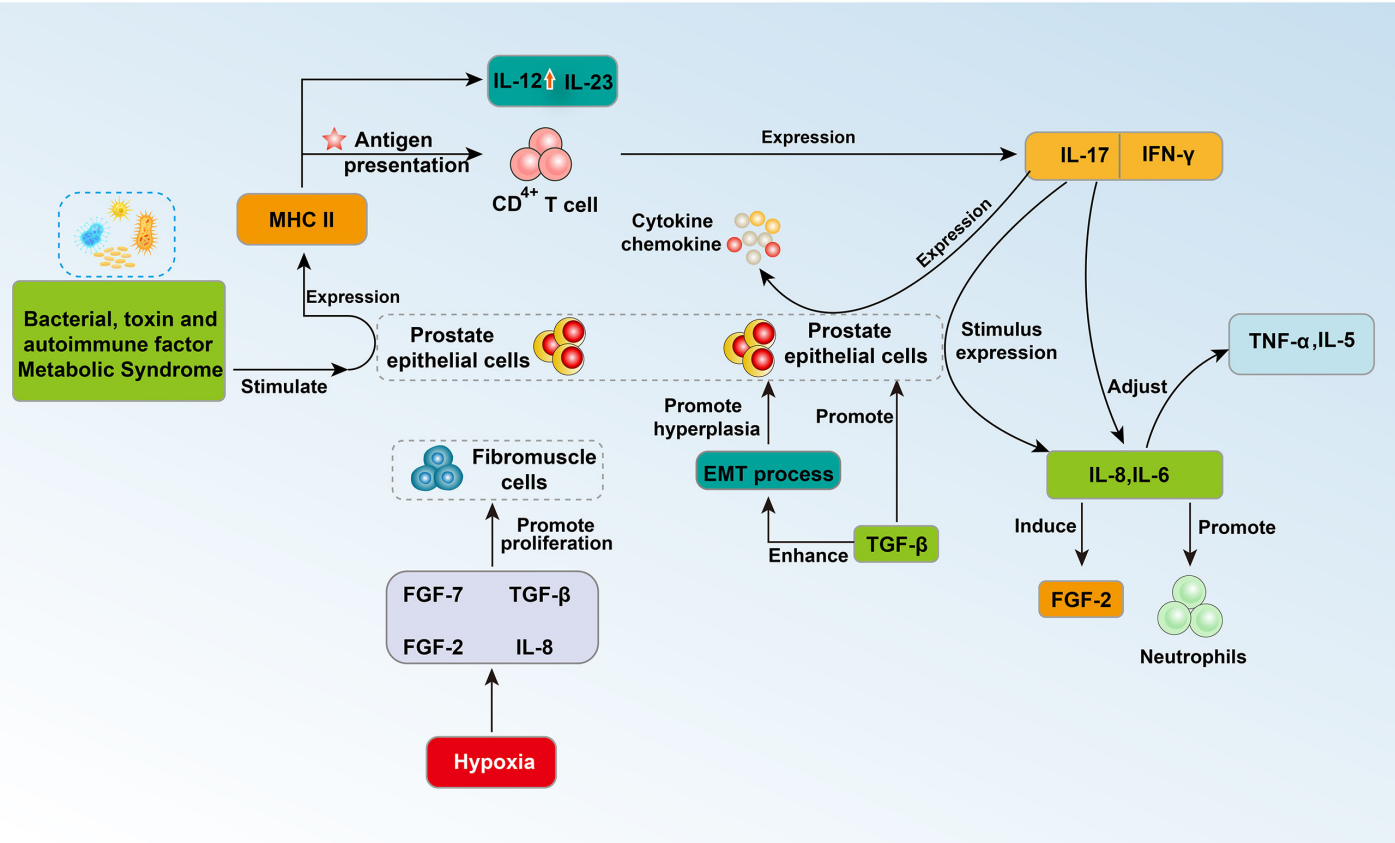
Schematic diagram of the interaction between prostate cells and related cytokines 1.

Insulin is reported to have mitogenic activity (22). Previous experiments have shown that the growth of the prostate requires insulin, and hyperinsulinemia can promote cell proliferation and prostate enlargement (23). Insulin-like growth factor-1 (IGF-1) has been shown to have a strong mitogenic and antiapoptotic effect on prostate tissue (24). Insulin-like growth factor binding protein-3 (IGFBP-3) regulates the interaction between IGF-1 and its receptor and was found to lower the level of IGF-1 and inhibit the anti-apoptotic properties of IGF-1 (25, 26) (**Figure 1**). Recent multivariate analysis (27) showed that insulin (p = 0.001) and IGFBP-3/PSA (p = 0.004) predict prostate size in patients with BPH. Insulin was increased and IGFBP-3/PSA was reduced in BPH patients with increased prostate size. Using a cut-off concentration of 527.52, the investigators found that the ratio of IGFBP-3/PSA in BPH tissue was distributed with 96% sensitivity and 96% specificity compared to normal prostate. The above evidence shows that as the size of the prostate increases, the patient’s endogenous insulin content increases, while IGFBP-3/PSA decreases (27) (**Figure 1**). This seems to indicate that through early intervention of the patient’s insulin content, the patient’s prostate size is more likely to be effectively controlled, which may provide the possibility to open up new methods for BPH management (**Figure 1**).

As a subtype of macrophages, M2 macrophages are the main inflammatory cells that infiltrate and proliferate the prostate. They mainly secrete cytokines and growth factors to promote the disease process of benign prostatic hyperplasia (28). Recently, based on human prostate tissues, prostate, and monocyte cell lines (WPMY-1, BPH-1, and THP-1), researchers have examined prostate cell proliferation, apoptosis, the cell cycle, epithelial-mesenchymal transition (EMT) and the process of fibrosis to explore the influence of M2a macrophages in the disease process of BPH (29). The results showed that M2a macrophages expressed more IGF-1 than other subtypes (29) (**Figure 1**). Knockdown of IGF-1 in WPMY-1 and BPH-1 cells attenuated cell proliferation, promoted cell apoptosis, retarded the cell cycle at the G0/G1 phase, and suppressed the EMT process in BPH-1 cells as well as the fibrotic process in WPMY-1 cells, which was reversible when cocultured with M2a macrophages (29). These data demonstrated that knockdown of IGF-1 expression in cultured BPH epithelial and stromal cells reduces proliferation and increases apoptosis. These effects are reversed by coculture with M2a macrophages (29). This indicates the direction for the precise treatment of BPH in the future (**Figure 1**).

Macrophage migration inhibitory factor (MIF) is a proinflammatory cytokine. Previous studies have found that MIF is highly expressed in the epithelium of BPH, which reveals the potential connection between MIF and BPH. Recently, Song and his colleagues discovered that recombinant human MIF (rMIF) promoted the proliferation of BPH-1 and PWR-1E cells, while ISO-1 (MIF antagonists) partially reversed this effect on proliferation (**Figure 1**). JC-1 assays showed that rMIF inhibited the apoptosis of BPH-1 and PWR-1E cells, and ISO-1 partially reversed this inhibition. Moreover, western blotting indicated that rMIF downregulated P53 and upregulated COX-2 (**Figure 1**). Furthermore, MIF-induced proliferation could be inhibited by celecoxib in the CCK8 and flow cytometry assays. MIF-inhibited apoptosis could be partially reversed by celecoxib in the JC-1 assay (**Figure 1**). Western blotting showed that celecoxib could partially reverse MIF-induced COX-2 upregulation and P53 downregulation. Together, MIF is highly expressed in BPH epithelium. *In vitro*, MIF promoted BPH epithelial cell growth by regulating COX-2 and P53 signaling (30) (**Figure 1**).

**Figure 2:**
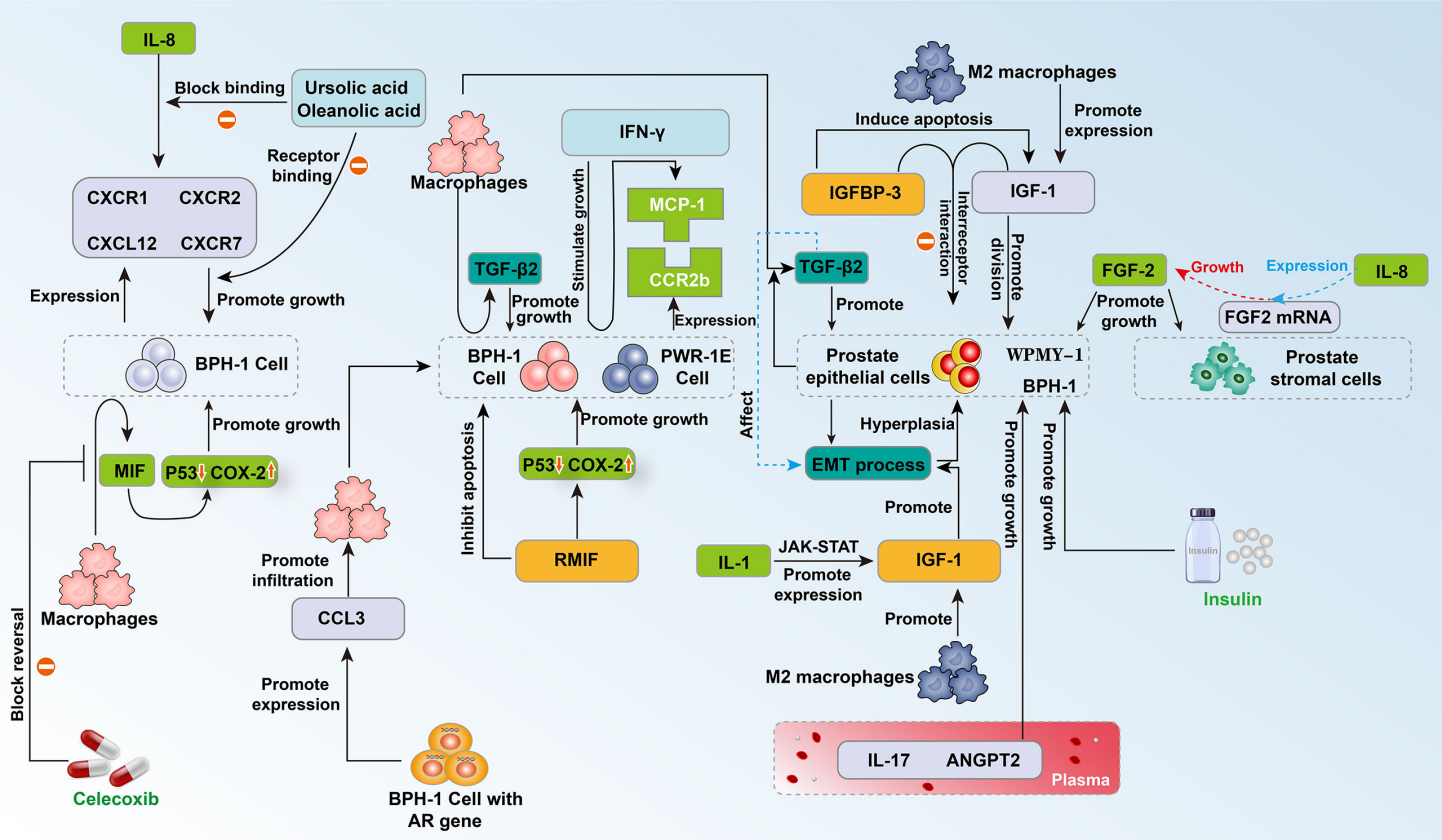
Schematic diagram of the interaction between prostate cells and related cytokines 2.

With the progression of inflammation, macrophages, IL-8, IL-1 and other inflammation-related substances accumulate locally, which can aggravate inflammation while increasing the growth rate of cells. BPH cells can secrete monocyte chemotactic protein-1 (MCP-1) under the stimulation of interferon-γ (IFN-γ). Western blotting showed that almost all prostate cells express the MCP-1 receptor CCR2. When MCP-1 combines with one subtype of its receptor, CCR2b, the proliferation of epithelial cells is promoted (31) (**Figure 1**). In addition, macrophages can induce the epithelial cells of the prostate tissue to express a large number of epithelial-mesenchymal transition factors, including transforming growth factor-β2 (TGF-β2), and cause the prostate to enlarge (32). Fibroblast growth factor 2 (FGF2) is an effective growth factor for prostate stromal cells and epithelial cells. IL-8 increases the level of FGF2 mRNA in prostate stromal cells, promotes the expression of FGF2, and causes abnormal cell proliferation in the transition zone of the prostate (33) (**Figure 1**). In addition, IL-8 can induce fibroblasts in prostate stromal nodules to show significant myosin aggregation and smooth muscle alpha-actin immune activity, which changes the biological activity of these cells and may cause abnormal proliferation (34). IL-1 can activate the Jak-STAT signaling pathway and stimulate the transcription and secretion of insulin-like growth factors (33, 35) (**Figures 1**, **2**).

While inflammation promotes the occurrence and development of BPH, the presence of BPH cells can also promote the occurrence of inflammation and make inflammation continue to expand. BPH cells can expand the inflammatory response by inducing more inflammatory cells to participate in inflammation, and this process can be achieved through many processes. For example, a comparative study found that BPH stromal cells retaining the AR gene can express a larger amount of inflammatory factor C-C motif chemokine 3 (CCL3) than BPH cells with AR gene knockout, which further induces the infiltration of macrophages (36). In addition, an enzyme-linked immunosorbent assay confirmed that prostate cells can secrete MCP-1 under the stimulation of IFN-γ, which induces the infiltration of macrophages and secretion of inflammatory factors (31) (**Figure 1**).

The latest research also shows that BPH may be an immunoinflammatory disease (37). The prostate is an organ with immune activity, and its internal immune system is developed and complex. Approximately 90% of prostate immune cells are T lymphocytes (38). In particular, CD8+ T lymphocytes are normally located in the periglandular area around epithelial ducts and between epithelial cells, whereas lymphoid aggregates that consist of B lymphocytes and parafollicular T lymphocytes are located in the fibromuscular stroma (12). Stimulating factors from different sources will activate the corresponding molecular pathways, leading to immune inflammatory changes in the prostate, which further affects the changes in prostate tissue structure, epithelial cells and prostate interstitial changes (28). Bacterial or viral infections, sexually transmitted diseases, autoimmune factors, and metabolic syndrome can transform the prostate into a proinflammatory state. The presence of these stimulating factors destroys prostate cells, leading to chronic inflammation (38). During the immune inflammatory reaction, a large number of lymphocytes infiltrate the glands, tissues around the glands and the stromal area, and the number of T lymphocytes continues to increase (19). These cells promote the release of cytokines and growth factors, which further leads to abnormal remodeling of the prostate structure characterized by tissue damage, chronic immune response, and fibromuscular growth (12, 19). The immune disorder response in BPH may occur through increased expression of proinflammatory IL-17, and the autoimmune response associated with T cells may induce abnormal proliferation of epithelial cells and stromal cells (**Figure 2**). These BPH cells further express MHC class II molecules, upregulate IL-12 and IL-23 subunits, and present antigens to CD4+ T cells. Additionally, BPH cells induce the secretion of IFN-γ and IL-17. The combination of IL-17 and IFN-γ seems to have a synergistic effect, further promoting the production of proinflammatory cytokines and chemokines in BPH cells and secreting IL-8 and IL-6. The process of IL-8 production is related to neutrophils in prostate tissue (39). In particular, interleukin-17 (IL-17) regulates the expression of IL-6 and IL-8 in epithelial cells and stromal cells and triggers the release of TNFa, IL-5 and other cytokines (40) (**Figure 2**).The release of IL-8 further induces the expression of fibroblast growth factor (FGF)-2, and FGF-2 can strongly promote the abnormal proliferation of prostate cells. Another clear relationship between inflammation and BPH is the expression of transforming growth factor β (TGF-β) (28). The expression level of this growth factor is elevated in the prostate with immune inflammation and may be involved in the epithelial-mesenchymal transition process (EMT) (28). Local hypoxia may have an effect on fibroblast-myofibroblast differentiation by promoting the formation of new blood vessels and the release of growth factors (such as FGF-7, TGF-b, FGF-2 and IL-8) (28) (**Figure 2**).

IL-6 and IL-8 in prostatic fluid are important cytokines that mediate the immune inflammation of prostate hyperplasia. Recently, researchers have studied the correlation between serum IL-6 (sIL-6) and serum IL-8 (sIL-8) in plasma and the occurrence of acute urinary retention (AUR) in patients with BPH (41–43) (**Figure 2**). The researchers used univariate and multivariate logistic regression analyses to conduct a cross-sectional study of sIL-6 and sIL-8 in 256 and 245 BPH patients and found that the levels of sIL-6 and sIL-8 in the AUR group were positively correlated with AUR. The odds ratios were [OR =1.365, 95% confidence interval (CI): 1.174–1.586, P<0.001] and [OR = 1.024, 95% CI: 1.009– 1.040, p = 0.002] (44, 45). The significance of these studies is to expose potential treatment sites for BPH patients with immunological inflammation, to screen high-risk groups of AUR through blood tests, to provide patients with early intervention opportunities, to further improve the prognosis and to improve the quality of life of patients.

In the study of Dinandra and his colleagues, the IL-8 axis was defined as IL-8, IL-8 receptor CXCR1 (R1), CXCR2 (R2), and CXCL12 receptor CXCR7 (R7), and CRISPR/Cas9 gene editing was used to evaluate the functional role of the IL-8 axis in the growth of prostate epithelial cells (**Figure 1**). Knockout of CXCR7 by gene editing reduces the expression of IL-8 and CXCR1 by 4- to 10-fold and inhibits the growth of prostate epithelial cell lines (BPH-1 cells) by greater than or equal to 50%. This finding suggests that IL-8 and its receptor components directly promote the growth of prostate epithelial cells through the IL-8 axis. Further studies have found that the combination of low-dose oleanolic acid (OA) and ursolic acid (UA) synergistically inhibits the growth of BPH epithelial cells. Under the lower drug index of UA and OA, the combination still has a strong blocking effect on the growth of prostate epithelial cells. The IL-8 axis is a potential promoter for the pathogenesis of BPH. By blocking this axis, the growth of BPH epithelial cells seems to be effectively inhibited, which requires further studies (46) (**Figure 1**).

In addition, interleukin-17 and angiopoietin-2 (ANGPT2) are important markers of inflammation and angiogenesis (47, 48). To explore the relationship between BPH and these two factors, the researchers measured the levels of IL-17 and ANGPT2 in BPH patients and normal controls. The results of the study found that the serum IL-17 and ANGPT2 levels of BPH patients were significantly higher than those of the normal control group (49). Multivariate analysis showed that ANGPT2 levels can be used to predict prostate size in BPH patients. Linear regression analysis indicated that IL-17 and ANGPT2 have a significant correlation in BPH patients. This study proved that serum IL-17 and ANGPT2 levels are potential biomarkers for predicting prostate size in BPH patients (49).

## The Relationship and Mutual Effect of Inflammation and Androgens in BPH

Existing clinical studies have proven that both androgens and inflammation can trigger the balance of cell proliferation and apoptosis in the prostate area. Regarding the effect on BPH, androgens and inflammation have a mutual influence.

Dihydrotestosterone is the main androgen in the prostate and acts through AR. Researchers have found that in 105 prostate resection samples, prostate volume and androgen receptor (AR) expression are significantly correlated with immune-mediated inflammation (15). Immune-mediated inflammation specimens have larger prostates and higher AR expression levels. This shows that the androgen pathway of the prostate can be affected by immune cells and proinflammatory cytokines. It also acts synergistically with hormones to enhance the proliferation and stimulation of prostate cells. However, whether there is a clear causal relationship between inflammation and AR expression requires further exploration (15). Some researchers have conducted experiments and inferred the above mechanism. IL-6 is produced in the inflammatory response, and IL-6 increases the level of prostate-specific antigen (PSA) mRNA, upregulates the expression of AR, and then regulates the androgen response pathway (15).

At the same time, testosterone has an immunosuppressive effect, and testosterone reduces the expression of innate immune molecules, thereby reducing the antibacterial ability of the rat prostate. Both epithelial and stromal cells of the prostate can upregulate proinflammatory signals and trigger an inflammatory response after bacterial challenge. Androgen-depleted animals had lower bacterial counts and few histological signs of inflammation than control groups (50). Researchers such as Quintar used rats with orchiectomy as samples and found that the expression of immune-related proteins (such as TLR4, CD14, MyD88, etc.) in the prostate gradually increased (50). It has been shown by immunocytochemical analysis that the expression of antibacterial proteins, such as rBD-1 and SP-D, increases (50). In addition, five days after E. coli was inoculated in the prostate area, the amount of bacteria in the prostate of the orchiectomy group was remarkably lower than that of the control group. Androgens play a certain role in the immunity of the prostate. Androgens may inhibit the occurrence and development of inflammation, thereby reducing the risk of further development of BPH.

## Inflammations as Targets for Prevention and Treatment

Considering the many pathways by which inflammation affects the occurrence and development of BPH, anti-inflammatory drugs can block or reduce abnormal tissue proliferation caused by inflammation at different points of the inflammatory pathway and are considered to be a possible way to prevent and treat BPH. Some researchers believe that prostate tissue damage can cause proliferative inflammatory atrophy (PIA), increase the expression level of cyclooxygenase-2 (COX-2), and indirectly lead to abnormal cell proliferation. Aspirin and nonaspirin nonsteroidal anti-inflammatory drugs (NSAIDs) reduce inflammation by inhibiting cyclooxygenase (COXs) (51, 52). Existing documents have reviewed the application effects of these drugs and believe that they inhibit abnormal cell proliferation and can even inhibit the progression of cancer. In addition, COX inhibitors may have the potential to control abnormal cell proliferation (53, 54). For example, COX-2-selective inhibitors can reduce the growth of prostate cancer cell lines, but their specific role is not clear (55).

Some population-based studies have shown that the use of aspirin is negatively correlated with the occurrence of prostate tissue hyperplasia and prostate cancer. If aspirin is used more frequently and for a longer period of time, the negative correlation seems to be greater. There are still some unresolved problems in the use of such drugs (56–58). For example, pharmacological evidence shows that hydrophobicity, acidity, alkalinity, and metabolic factors can affect the tendency of drugs to enter prostate tissue (59). If the drugs cannot accumulate to an effective concentration, it may reduce the effect or even be invalid. The relationship between nonaspirin steroids and the control of abnormal hyperplasia of prostate tissue is very different, there is usually no clear statistical significance, and the results are usually mixed (56, 60, 61).

In inflammation caused by bacterial infection, antimicrobials are often the first choice. Antibacterial therapy requires a drug to reach a certain concentration, and its success depends on the drug’s antibacterial activity and pharmacokinetic characteristics (62, 63). In the treatment of chronic inflammation, phytotherapy, such as pollen extract in combination with other drugs, is also an approach. For inflammation caused by nonbacterial infection, in daily life, improving diet and physical exercise can regulate the composition of intestinal flora, affect the body’s metabolism and immunity, and delay the disease (62).

Based on the existing research and taking into account the side effects of the drug and the tissue, the combination of anti-inflammatory drugs and cell markers can achieve targeted therapy, which is most likely to improve the efficiency of treatment and effectively avoid risks. This study will provide new ideas for the clinical treatment of BPH and has positive application prospects.

## Conclusions

In summary, inflammation plays a vital role in the pathogenesis and disease development of BPH. Inflammation can independently affect the development of BPH in a variety of ways, and it can also interact with androgens. At the same time, BPH can also promote inflammation to expand its scope and aggravate the situation. Therefore, in the treatment process, early intervention in the occurrence and development of inflammation in prostate tissue can slow down the progression of BPH. The combination of standard therapies and anti-inflammatory measures may provide valuable new ideas for the treatment of BPH. In addition, because BPH and prostate cancer have very similar histological, pathological and genetic characteristics, the control of inflammation in prostate disease is likely to provide a potential treatment for prostate cancer.

## Author Contributions

The conceived and designed the experiments: QW and LL. Analyzed the data: DC and RS. Contributed reagents/materials/analysis: LP, JZL, YH, ZC, BC, JL, JA, and LY. All authors contributed to the article and approved the submitted version.

## Funding

This study was funded by the National Natural Science Foundation of China (Grant Number 82000721) and Programs from Department of Science and Technology of Sichuan Province (Grant Number 2020YJ0054).

## Conflict of Interest

The authors declare that the research was conducted in the absence of any commercial or financial relationships that could be construed as a potential conflict of interest.

## Publisher’s Note

All claims expressed in this article are solely those of the authors and do not necessarily represent those of their affiliated organizations, or those of the publisher, the editors and the reviewers. Any product that may be evaluated in this article, or claim that may be made by its manufacturer, is not guaranteed or endorsed by the publisher.
